# Prognostic nomogram for heat stroke patients based on rapidly accessible clinical indicators

**DOI:** 10.3389/fmed.2025.1603374

**Published:** 2025-07-25

**Authors:** Tianshan Zhang, Bojie Xiao, Guo Tang, Tao Cheng, Hongguang Gao, Ping Zhang, Rong Yao

**Affiliations:** ^1^Emergency Department of West China Hospital, Sichuan University, Chengdu, China; ^2^Department of Emergency Medicine, West China Hospital, West China School of Nursing, Sichuan University, Chengdu, China

**Keywords:** heat stroke, in-hospital mortality, emergency department, nomogram, multiorgan damage

## Abstract

**Purpose:**

To develop and validate a rapid-assessment scoring system for predicting in-hospital mortality in heat stroke (HS) patients, thereby facilitating early identification and intervention for critical cases.

**Approach:**

We conducted a retrospective cohort analysis of HS patients admitted to emergency department (ED) of 13 hospitals in southwest of China between July 1, 2022 and December 31, 2024. Clinical parameters including demographic data, initial vital signs, and major organ function biomarkers were systematically collected. Patients were further divided into a training cohort and a validation cohort at a 7:3 ratio. The primary endpoint was all-cause in-hospital mortality. Through rigorous variable selection using Least Absolute Shrinkage and Selection Operator (LASSO) regression followed by multivariable logistic regression modeling, we developed a prognostic nomogram. Model performance was assessed via receiver operating characteristic (ROC) curve analysis, decision curve analysis (DCA), and clinical impact curve (CIC) evaluation, with comparative benchmarking against established scoring systems [Sequential Organ Failure Assessment (SOFA) and Acute Physiology and Chronic Health Evaluation II (APACHE II)].

**Findings:**

A total of 307 patients were included in the study. 114 experienced in-hospital mortality, while 193 survived. Non-survivors exhibited significantly altered baseline values across multiple physiological domains: reduced Glasgow Coma Scale (GCS), impaired oxygenation index (OI), elevated fibrin degradation products (FDP), D-dimer, activated partial thromboplastin time (APTT), and serum creatinine (Cr) (all *p* < 0.0001). Through LASSO regression followed by multivariate logistic regression analysis, 27 initially significant variables were refined to four independent prognostic indicators: Cr, GCS, OI, and FDP. These predictors were subsequently integrated into a multivariate prognostic nomogram demonstrating discriminative capacity for mortality risk stratification in both training (AUC 0.811, 95% CI 0.751–0.871) and validation cohorts (AUC 0.766, 95% CI 0.706–0.826). DCA revealed superior net benefit across clinically relevant probability thresholds. The AUC of the nomogram in the entire cohort (0.794) was significantly superior to the SOFA score (0.703, DeLong’s test, *p* = 0.0008) and comparable to the APACHE II score (0.765, DeLong’s test, *p* = 0.3581).

**Conclusion:**

We developed and validated a prognostic tool utilizing routinely available parameters in ED to predict in-hospital mortality in HS patients. This clinically implementable model demonstrates comparable accuracy to established intensive care scoring systems while offering distinct advantages in rapid bedside application, potentially enabling time-critical therapeutic decisions in emergency settings.

## 1 Introduction

Heat stroke (HS) is a life-threatening clinical emergency characterized by environmental heat-induced thermoregulatory failure, rapid core temperature elevation (>40°C), neurological dysfunction, and concurrent multi-organ dysfunction syndrome (MODS) (1). The escalating frequency of extreme heat events, exacerbated by anthropogenic climate change, has precipitated a concerning surge in heat-related morbidity, positioning HS as a critical public health challenge of the human society (2). Etiological classification distinguishes between classical heat stroke (CHS) and exertional heat stroke (EHS), reflecting distinct pathogenic mechanisms and population susceptibilities (3). CHS manifests as passive thermoregulatory collapse in vulnerable demographics during environmental heat extremes, whereas EHS emerges from metabolic overdrive during strenuous exertion in otherwise healthy individuals (4).

HS induces rapid multi-organ catastrophe, with pathognomonic progression to MODS within hours of onset through coordinated assault on cerebral, cardiovascular, pulmonary, hepatobiliary, renal, and gastrointestinal systems (5–7). Contemporary pathophysiological models propose a dual-pathway mechanism: Direct thermal cytotoxicity from supra-physiological core temperatures and systemic inflammatory cascade involving heat stress-induced endotoxemia, systemic inflammatory response syndrome (SIRS) (8). Experimental models demonstrate HS-induced endothelial barrier disintegration, characterized by ultrastructural damage to vascular endothelia in cardiopulmonary, hepatic, and splanchnic circulations, precipitating consumptive coagulopathy and sterile inflammation (9–13).

There have been some advances in the pathogenesis of HS. However, current therapeutic strategies for HS are still predominantly anchored in generic intensive care scoring systems, notably the Sequential Organ Failure Assessment (SOFA) (14) and Acute Physiology and Chronic Health Evaluation II (APACHEII) (15). These established frameworks exhibit critical limitations when applied to HS populations: their inability to distill disease-specific prognostic indicators from comprehensive admission datasets, while their multi-parameter acquisition protocols introduce operational complexities ill-suited to emergency workflows. SOFA and APACHE-II require complex calculations and 12–24 variables causing delays impractical for ED triage. Their non-specificity to HS pathophysiology further limits prognostic accuracy. Moreover, the absence of validated HS-specific stratification tools precludes precise quantification of baseline multi-organ injury burden. The lack of validated HS-specific stratification tools precludes precise quantification of multi-organ injury burden at baseline. Wang et al. (16) conducted a retrospective analysis of 70 HS cases, identifying procalcitonin (PCT), platelet count, and D-dimer levels as potential biomarkers for HS stratification. In a cohort study involving 151 HS patients, Chen et al. (17) established significant correlations between standard base excess (SBE) values and clinical outcomes. Concurrently, Tang et al. (18) proposed troponin I levels at ICU admission as a prognostic indicator through their analysis of 67 critical HS cases. The above studies have proved that some characteristic data can be used to predict the prognosis of patients in HS. Nevertheless, prognostic assessment tools for ED triage of HS patients remain critically underdeveloped, with current literature constrained by single-center designs and insufficient validation of rapid-assessment parameters - a critical gap impeding early identification of high-risk patients and timely intervention escalation.

The aim of this study is to identify the risk factors associated with mortality in patients with HS and to develop a clinically implementable predictive model that integrates cost-effective, readily obtainable parameters for real-time mortality risk stratification and provide emergency clinicians with a rapid decision-making tool.

## 2 Methods

This retrospective observational study analyzed the clinical data of patients diagnosed with HS who were admitted to the ED of 13 hospitals in southwest China between July 1, 2022, and December 31, 2024. The study focused on both classic and exertional forms of HS, defined by the triad of hyperthermia, neurologic abnormalities, and recent exposure to hot weather or physical exertion (4). Patients were excluded if they met any of the following criteria: (1) Patients younger than 14 years; (2) Patients lack of medical histories and blood test; (3) Patients coexisting with other irreversible diseases affecting mortality.

For each patient, a detailed clinical profile was collected. This included medical histories, time from symptom onset to presentation (time-to-ED), physiological parameters and laboratory test results on the first day arriving at ED. Additionally, Glasgow Coma Scale (GCS), SOFA, and APACHE II scores were calculated using clinical and laboratory data. All data were retrieved from hospital electronic medical records. The primary outcome of the study was the survival status of patients at hospital discharge.

The experimental protocol was approved by the Biomedical Ethics Review Committee of West China Hospital, Sichuan University (No. 2022–1,478) in accordance with the Declaration of Helsinki. Informed consent was waived by the Biomedical Ethics Review Committee of West China Hospital, Sichuan University (No. 2022–1,478) due to the retrospective nature of this research.

### 2.1 Statistical analysis

All statistical analyses were performed using R software (version 4.3.1; R Foundation for Statistical Computing). Missing data handling was implemented through multiple imputation techniques utilizing the “mice” package (version 3.16.0), generating 50 complete datasets through chained equations to preserve statistical power and mitigate bias. The imputation process incorporated predictive mean matching with proper consideration of variable distributional characteristics. Final estimates were obtained using Rubin’s rules for pooling results, thereby accounting for between-imputation variance. Among all covariates, missing data proportions ranged from 5.21 to 11.40%.

The dataset underwent systematic partitioning into training and validation subsets at a 7:3 ratio, with this stratification process being executed through the implementation of a stratified resampling technique to preserve distributional parity of survival outcomes across both cohorts. Continuous variables were characterized using appropriate measures of central tendency and dispersion: normally distributed parameters were summarized as mean ± standard deviation (SD) and compared via Student’s t-test with Welch’s correction for unequal variances, while nonparametric variables were expressed as median interquartile range (IQR) [M(IQR)], and analyzed using Mann–Whitney U tests with exact permutation implementation. Categorical variables were presented as frequency counts with percentages (n, %) and assessed through Pearson’s *χ*^2^ test or Fisher’s exact test as appropriate based on expected cell frequencies.

We performed Least Absolute Shrinkage and Selection Operator (LASSO) regression with 10-fold cross-validation using the “glmnet” package, applying the one standard error (1-SE) criterion for optimal lambda selection. The selected predictors were subsequently incorporated into multivariate logistic regression models through the forced entry method utilizing the “rms” package. Results were expressed as odds ratios (ORs) with corresponding 95% confidence intervals (CIs). Model performance was compared against established prognostic scoring systems including the Sequential Organ Failure Assessment (SOFA) and Acute Physiology and Chronic Health Evaluation II (APACHE II). Discrimination capacity was evaluated via receiver operating characteristic (ROC) curve analysis using the “pROC “package, with the area under the curve (AUC) quantifying predictive accuracy.

The optimal prediction model was transformed into a clinical decision tool using nomogram construction via the “rms” package. Nomogram points reflect weighted contributions based on multivariate coefficients. Calibration accuracy was assessed through: (1) calibration-in-the-large (intercept test), (2) calibration slopes via restricted cubic splines (3 knots), and (3) graphical calibration plots with 45° reference lines. Internal validation was performed on a 30% holdout sample (stratified by outcome) using standard ROC analysis. All statistical tests were two-tailed with *α* = 0.05 significance threshold. The nomogram was assessed using Calibration curves, decision curve analysis (DCA), and clinical impact curves (CIC) using the “rmda “package for both the training and validation cohorts.

## 3 Results

### 3.1 Study population and univariate analysis

**Figure 1:**
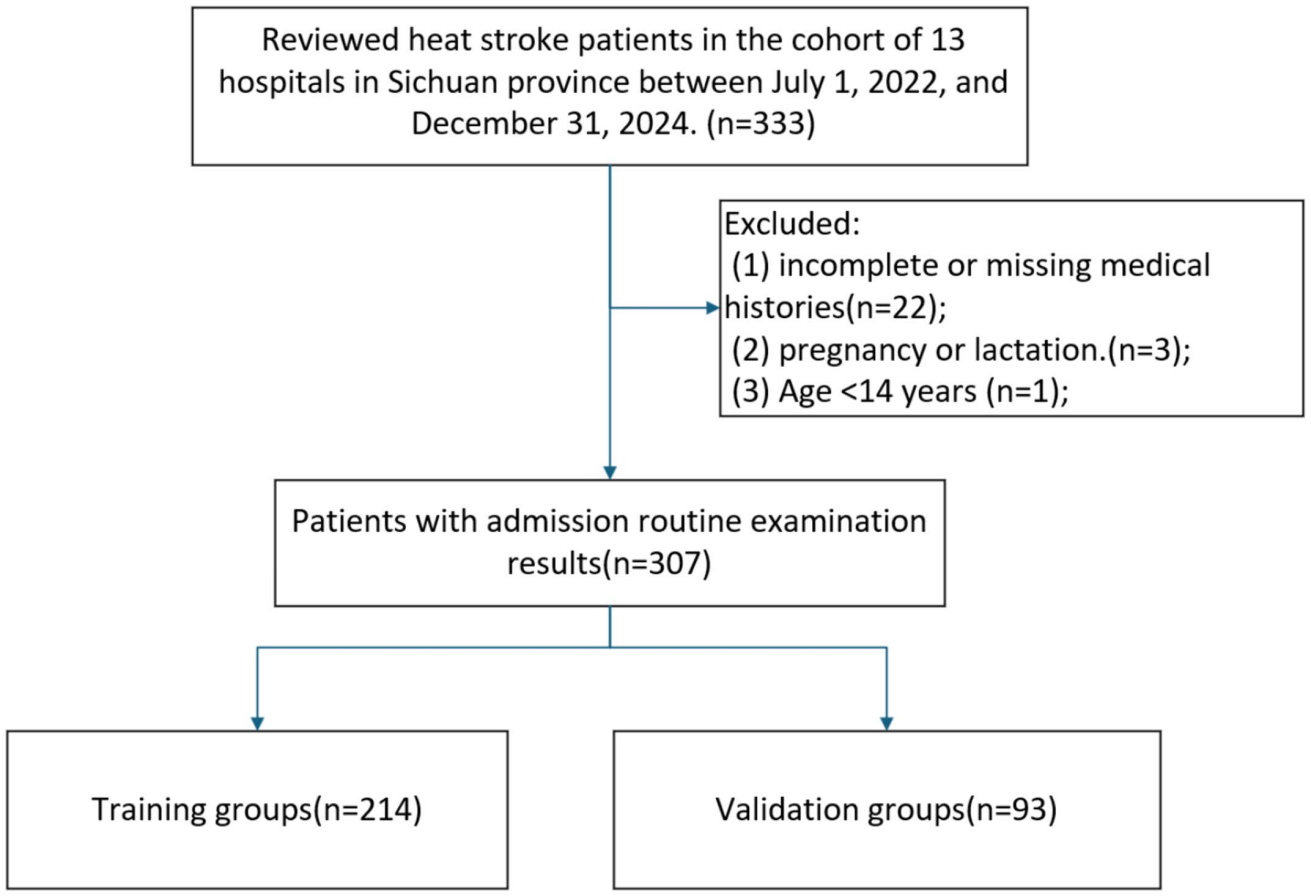
The flowchart of this study.

The study cohort comprised 307 HS patients meeting inclusion criteria, detailed information was shown in Figure 1. The median age of the patients was 65 years (IQR: 52–75). Among the included patients, 144 (46.91%) were female, while the majority (53.09%) were male. 114 patients (37.13%) experienced in-hospital mortality, while 193 survived. 214 patients were assigned to the training cohort and 93 to the validation cohort with a proportion of 7:3. No statistically significant differences were observed in baseline characteristics between the training and validation groups, Table 1.

**Table 1:** Baseline characteristics of the entire HS cohort (Training + Validation).

Variables	Category	Overall	Validation group	Training group	*p*
n		307	93	214	
Classification (%)	EHS	113 (36.69)	36 (38.71)	77 (35.81)	0.7439
	CHS	194 (63.19)	57 (61.29)	137 (64.02)	
Time_to_ED (hour)		7.00 [3.00, 48.00]	10.00 [3.00, 48.00]	7.00 [2.50, 48.00]	0.6449
Age (year)		65.00 [51.60, 75.00]	65.00 [51.00, 75.00]	65.90 [52.00, 74.75]	0.6680
Gender (%)	Male	163 (52.92)	52 (55.91)	111 (51.63)	0.5704
	Female	144 (47.08)	41 (44.09)	104 (48.37)	
Outcome (%)	Survival	193 (62.87)	58 (62.37)	135 (63.08)	0.9796
	Death	114 (37.13)	35 (37.63)	79 (36.92)	
T (°C)		38.80 [37.05, 40.35]	39.00 [36.90, 40.00]	38.60 [37.20, 40.40]	0.825
HR (beats/min)		111.00 [93.50, 131.00]	113.00 [93.00, 130.00]	110.50 [94.00, 132.00]	0.7941
RR (breaths/min)		22.00 [20.00, 27.00]	23.00 [20.00, 28.00]	22.00 [20.00, 26.00]	0.1829
SBP (mmHg)		121.00 [101.50, 140.00]	124.00 [100.00, 148.00]	120.00 [102.00, 135.00]	0.2822
DBP (mmHg)		72.00 [60.00, 82.00]	74.00 [62.00, 82.00]	71.00 [59.25, 81.75]	0.3266
SpO_2_ (%)		96.00 [94.00, 98.00]	96.00 [94.00, 98.00]	96.00 [93.00, 98.00]	0.2371
GCS		8.00 [3.00, 12.00]	8.00 [3.00, 12.00]	7.50 [3.00, 12.00]	0.8335
SOFA		7.00 [4.00, 9.00]	7.00 [4.00, 10.00]	7.00 [5.00, 9.00]	0.4358
APACHE II		23.00 [17.00, 28.00]	23.00 [18.00, 29.00]	22.50 [17.00, 27.00]	0.3876

Time_to_ED, time from symptom onset to presentation; HR, Heart Rate; RR, Respiratory Rate; SBP, Systolic Blood Pressure; DBP, Diastolic Blood Pressure; SpO_2_, oxygen saturation in arterial blood; GCS, Glasgow Coma Scale; SOFA, Sequential Organ Failure Assessment; APACHE II, Chronic Health Evaluation II.

In training group, non-survivor patients were older and have lower GCS scores [5.00(3.00,8.00) vs. 9.00(5.00,13.50), *p* < 0.0001] compared with survivor group at admission. Comparative analysis revealed significant coagulation profile disparities between survival cohorts, with non-survivors demonstrating elevated fibrin degradation products [FDP: 13.50 (9.95, 37.85) vs. 10.10 (4.72, 11.60); *p* < 0.0001] and D-dimer levels [7.22 (2.72, 17.73) vs. 3.88 (1.31, 6.01); *p* < 0.0001]. Non-survivors exhibited pronounced coagulopathic derangements characterized by prolonged activated partial thromboplastin time [APTT: 28.50 (26.60, 37.05) s vs. 27.10 (24.50, 28.95) s; *p* = 0.0001] and impaired oxygenation capacity [OI: 284.40 (188.86, 288.60) vs. 289.25 (288.60, 315.50); *p* < 0.0001]. Concurrent renal dysfunction was evidenced by elevated creatinine levels [Cr: 142.30 (102.00, 220.70) vs. 91.00 (72.00, 126.50); *p* < 0.0001], suggesting multiorgan failure as a terminal pathway in fatal HS cases. Besides, there was a significant difference between the non-survivor and survivors groups in heart rate, core temperature, lymphocyte count, basophil count, Cystatin C, Uric acid, BUN, direct bilirubin, aspartate aminotransferase, myoglobin, creatine kinase, lactate dehydrogenase, serum lactate, ALB, LDL, Ca, BE, PT, TT and so on (all *p* < 0.05; Table 1). Variables demonstrating statistically significant differences (*p* < 0.05) between groups in training cohort were included in subsequent data analysis detailed baseline characteristics of the patients can be found in Table 2.

**Table 2:** Characteristics of the HS training cohort stratified by in-hospital mortality outcome, including additional biomarkers from univariate screening.

Variables	Category	Overall	Survival	Non-survival	*p*
n		214	135	79	
Classification (%)	EHS	77 (35.98)	55 (40.74)	22 (27.85)	0.0803
	CHS	137 (64.02)	80 (59.26)	57 (72.15)	
Gender (%)	Male	111 (51.87)	67 (49.63)	44 (55.70)	0.4744
	Female	103 (48.13)	68 (50.37)	35 (44.30)	
Age (year)		65.90 [52.00, 74.75]	61.00 [49.00, 72.50]	70.00 [57.00, 76.00]	0.0097
T (°C)		38.60 [37.20, 40.40]	38.50 [37.00, 40.00]	39.20 [37.55, 40.75]	0.0197
MAP (mmHg)		89.33 [76.00, 101.45]	89.00 [77.30, 98.00]	91.00 [70.00, 105.50]	0.9918
Time_to_ED (hour)		7.00 [2.25, 48.00]	8.00 [2.00, 48.00]	6.00 [3.00, 63.50]	0.4381
HR (beats/min)		110.50 [94.00, 132.00]	107.00 [89.00, 125.50]	119.00 [98.00, 140.00]	0.0128
RR (breaths/min)		22.00 [20.00, 26.00]	22.00 [20.00, 26.00]	22.00 [20.00, 26.50]	0.2704
GCS		7.50 [3.00, 12.00]	9.00 [5.00, 13.50]	5.00 [3.00, 8.00]	<0.0001
PLT (10^9/L)		134.00 [86.00, 185.25]	145.00 [87.50, 206.00]	123.00 [79.50, 167.00]	0.1926
RBC (10^9/L)		4.12 [3.72, 4.53]	4.17 [3.74, 4.60]	4.06 [3.68, 4.45]	0.3038
HB (g/L)		125.00 [111.00, 138.00]	126.00 [111.50, 137.50]	123.00 [111.00, 138.00]	0.3359
HCT (%)		0.41 [0.35, 33.65]	0.41 [0.36, 31.80]	0.41 [0.34, 35.65]	0.7109
WBC (10^9/L)		10.94 [8.32, 14.61]	10.61 [8.32, 14.20]	11.62 [8.27, 15.18]	0.205
N (10^9/L)		8.95 [5.91, 12.30]	8.84 [6.36, 12.00]	9.06 [5.70, 13.02]	0.7688
L (10^9/L)		1.15 [0.62, 2.03]	1.00 [0.58, 1.73]	1.41 [0.77, 2.55]	0.0025
M (10^9/L)		0.65 [0.41, 0.98]	0.65 [0.41, 0.95]	0.65 [0.43, 1.04]	0.7349
EOS (10^9/L)		0.01 [0.00, 0.04]	0.01 [0.00, 0.04]	0.01 [0.00, 0.04]	0.0711
BASO (10^9/L)		0.02 [0.01, 0.04]	0.02 [0.01, 0.03]	0.03 [0.01, 0.05]	0.0291
TBiL (μmol/l)		18.00 [12.82, 24.20]	17.30 [12.05, 23.55]	19.20 [13.30, 25.00]	0.2415
DBiL (μmol/l)		7.05 [4.32, 11.16]	6.38 [4.04, 10.30]	8.10 [6.15, 11.64]	0.0028
ALT (IU/L)		41.00 [17.12, 89.75]	34.00 [18.00, 70.05]	52.00 [17.00, 135.00]	0.0803
AST (IU/L)		61.00 [34.00, 180.60]	48.00 [32.50, 141.00]	84.00 [38.00, 257.50]	0.0054
ALB (g/L)		38.25 [33.92, 41.88]	39.20 [34.90, 42.70]	36.00 [32.65, 39.55]	0.0002
Glu (mmol/L)		9.45 [6.96, 12.88]	8.95 [6.65, 12.29]	10.27 [7.17, 13.76]	0.0974
BUN (mmol/L)		7.53 [5.41, 10.20]	6.60 [5.00, 9.75]	8.80 [6.68, 12.41]	<0.0001
Cr (μmol/l)		105.20 [77.03, 151.80]	91.00 [72.00, 126.50]	142.30 [102.00, 220.70]	<0.0001
CysC (μmol/l)		1.20 [0.91, 1.39]	1.10 [0.84, 1.38]	1.38 [1.06, 1.71]	0.0002
Uric acid (mmol/L)		343.50 [242.75, 471.75]	314.00 [241.50, 425.90]	434.00 [269.50, 514.50]	0.0148
TG (mmol/L)		1.13 [0.69, 1.36]	1.18 [0.76, 1.41]	0.96 [0.67, 1.36]	0.1435
TC (mmol/L)		3.92 [3.31, 4.37]	3.94 [3.53, 4.52]	3.92 [3.02, 3.92]	0.0043
HDL (mmol/L)		1.02 [0.81, 1.17]	1.02 [0.82, 1.28]	1.01 [0.80, 1.13]	0.2943
LDL (mmol/L)		2.57 [1.94, 2.83]	2.57 [2.07, 2.89]	2.50 [1.77, 2.57]	0.026
LDH (IU/L)		377.00 [280.00, 520.00]	345.00 [262.50, 520.00]	483.00 [315.70, 550.50]	0.0009
PH		7.43 [7.38, 7.48]	7.42 [7.38, 7.48]	7.43 [7.35, 7.48]	0.1461
OI		288.60 [259.92, 294.65]	289.25 [288.60, 315.50]	284.40 [188.86, 288.60]	<0.0001
PCO_2_ (mmHg)		28.70 [23.80, 31.55]	28.85 [23.60, 31.30]	28.30 [24.40, 32.95]	0.3466
Lac (mmol/L)		2.97 [1.80, 3.70]	2.80 [1.70, 2.97]	3.10 [2.35, 5.50]	0.0001
Na (mmol/L)		133.80 [129.20, 139.07]	135.00 [129.75, 139.75]	132.60 [127.00, 137.85]	0.0909
K (mmol/L)		3.58 [3.08, 4.00]	3.59 [3.02, 3.91]	3.55 [3.17, 4.18]	0.3559
Cl (mmol/L)		101.20 [96.93, 106.65]	101.70 [97.00, 107.25]	101.20 [96.60, 106.00]	0.3559
Ca (mmol/L)		1.90 [1.06, 2.10]	1.95 [1.06, 2.12]	1.85 [1.05, 2.04]	0.0558
BE (mmol/L)		−4.98 [−7.50, −2.05]	−4.98 [−6.30, −1.90]	−5.00 [−8.85, −2.35]	0.021
HCO_3_ (mmol/L)		19.20 [15.95, 21.08]	19.35 [16.55, 21.15]	19.20 [15.55, 21.00]	0.4495
Mb (ng/ml)		704.70 [258.05, 3000.00]	704.70 [211.05, 1850.00]	2070.00 [470.51, 3000.00]	0.0014
CK (ng/ml)		737.65 [305.47, 3531.00]	610.00 [259.65, 2685.00]	1234.00 [448.15, 3531.00]	0.0093
CKMB (ng/ml)		6.61 [2.32, 34.49]	7.24 [2.32, 25.28]	6.14 [2.46, 42.16]	0.2065
cTnT (ng/ml)		30.70 [7.12, 157.00]	30.70 [8.35, 120.05]	30.70 [5.58, 366.00]	0.2266
BNP (pg/ml)		302.00 [141.25, 598.25]	306.00 [141.50, 576.00]	302.00 [127.50, 1151.00]	0.7465
PT (s)		12.60 [11.60, 14.55]	12.50 [11.20, 13.70]	13.60 [12.15, 15.70]	0.0001
INR		1.13 [1.05, 1.27]	1.13 [1.04, 1.22]	1.17 [1.10, 1.36]	0.0005
APTT (s)		27.40 [25.02, 30.90]	27.10 [24.50, 28.95]	28.50 [26.60, 37.05]	0.0001
TT (s)		18.30 [16.92, 19.80]	18.30 [16.85, 19.05]	18.50 [17.10, 21.90]	0.0308
FIB (g/L)		2.45 [1.88, 3.09]	2.45 [1.90, 3.09]	2.35 [1.83, 3.03]	0.2856
FDP (mg/L)		10.10 [5.71, 18.30]	10.10 [4.72, 11.60]	13.50 [9.95, 37.85]	<0.0001
D-dimer (mg/IFEU)		3.97 [1.65, 9.12]	3.88 [1.31, 6.01]	7.22 [2.72, 17.73]	<0.0001

PLT, Platelet Count; RBC, Red Blood Cell Count; HB, Hemoglobin; HCT, Hematocrit; WBC, White Blood Cell Count; N: Neutrophil Count; L: Lymphocyte Count; M: Monocyte Count; EOS, Eosinophilia Count; BASO, Basophil Count; TIBL, Total Bilirubin; DIBL, Direct Bilirubin; ALT, Alanine Aminotransferase; AST, Aspartate Aminotransferase; ALB, Albumin; Glu, Glucose; BUN, Blood Urea Nitrogen; Cr, Blood Creatinine; CysC, CystainC; TG, Triglyceride; TC, Total Cholesterol; HDL, High Density Lipoprotein Cholesterol; LDL, Low Density Lipoprotein Cholesterol; LDH, Lactate Dehydrogenase; OI, Oxygenation Index; PCO_2_, Partial Pressure of Carbon Dioxide; Lac, Serum Lactate; BE, Base Excess; HCO_3_, Serum Bicarbonate; Mb, Myoglobin; CK, Creatine Kinase; CKMB, Creatine Kinase Myocardial Band; cTnT, Cardiac Troponin I; BNP, Brain Natriuretic Peptide; PT, Prothrombin Time; INR, International Normalized Ratio; APTT, Activated Partial Thromboplastin Time; TT, Thrombin time; FIB, Fibrinogen; FDP, Fibrin Degradation Products.

### 3.2 Nomogram variable screening

**Figure 2:**
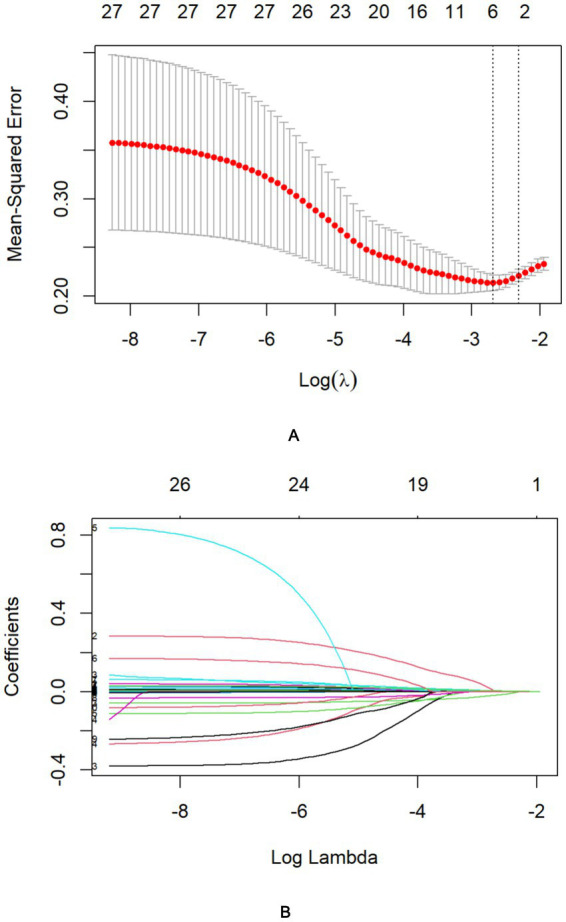
Identification of risk factors for in-hospital mortality in HS patients using LASSO regression with admission indices. **(A)** The best penalty coefficient l for Lasso regression is ascertained through 10-fold cross-validation and the one standard error (1SE) rule. **(B)** The LASSO regression coefficient plot for clinical features.

Following LASSO regression analysis, five variables (Cr, GCS, OI, cystatin C, and FDP) exhibiting non-zero coefficients were selected for model optimization based on clinical interpretability and parsimony principles. Although D-dimer and APTT showed univariate significance, LASSO regression excluded them due to high collinearity with FDP (reflecting shared coagulopathy pathways). FDP was retained for its superior specificity in diagnosing disseminated intravascular coagulation (19)—a critical HS complication—and its direct quantification of fibrinolysis, aligning with pathophysiological priority. These variables were subsequently incorporated into the final predictive model trained on the training cohort (Figure 2). After multivariable logistic regression analysis, GCS (OR: 0.8600, 95% CI: 0.7900–0.9300), Cr (1.0051, 1.0011–1.0091), OI (0.993, 0.9888–0.9972), and FDP (1.0200, 1.0000–1.0300) were identified as independent predictors of HS (Table 3).

**Table 3:** Logistic regression of risk factors for in-hospital mortality based on LASSO regression.

Variables	Adj. OR (95% CI)	*p*
GCS	0.86 (0.79,0.93)	<0.001
Cr	1.0051 (1.0011,1.0091)	0.003
OI	0.993 (0.9888,0.9972)	<0.001
FDP	1.02 (1,1.03)	0.011

Adj. OR, adjusted odds ratio.

**Figure 3:**
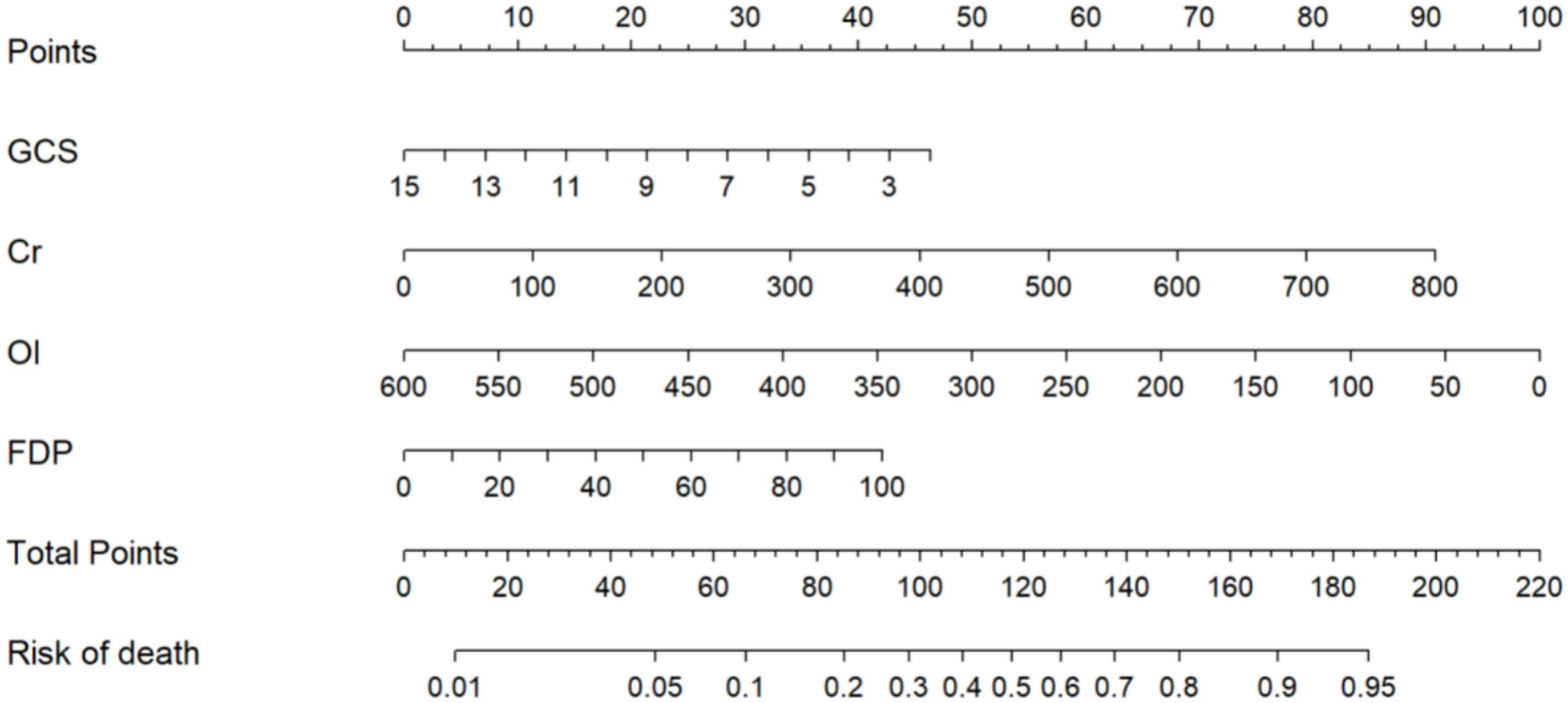
Nomogram for in-hospital mortality in patients with HS.

Consequently, these four prognostic determinants were integrated into a multivariate prediction algorithm, and a clinically oriented nomogram was subsequently developed to quantify individualized mortality probabilities through nonlinear transformations of independent risk predictors (Figure 3). Total scores (range: 0–220) are subsequently projected onto a mortality probability continuum at the nomogram base, enabling direct conversion of multidimensional clinical data into survival estimates. For clinical implementation, practitioners sequentially map each biomarker measurement to its corresponding point contribution via perpendicular axes intersection, then sum all component points to derive aggregate mortality risk. Clinically relevant risk strata: low (<20% mortality; <86 points), moderate (20–60%; 86–128 points), and high (>60%; >128 points)—enabling rapid decision pathways. Example calculation: Cr = 100 μmol/L (22 points), GCS = 7 (60 points), OI = 500 mmHg (36 points), FDP = 20 mg/L (18 points). Total = 136 points → Predicted mortality = 68% (high risk).

**Figure 4:**
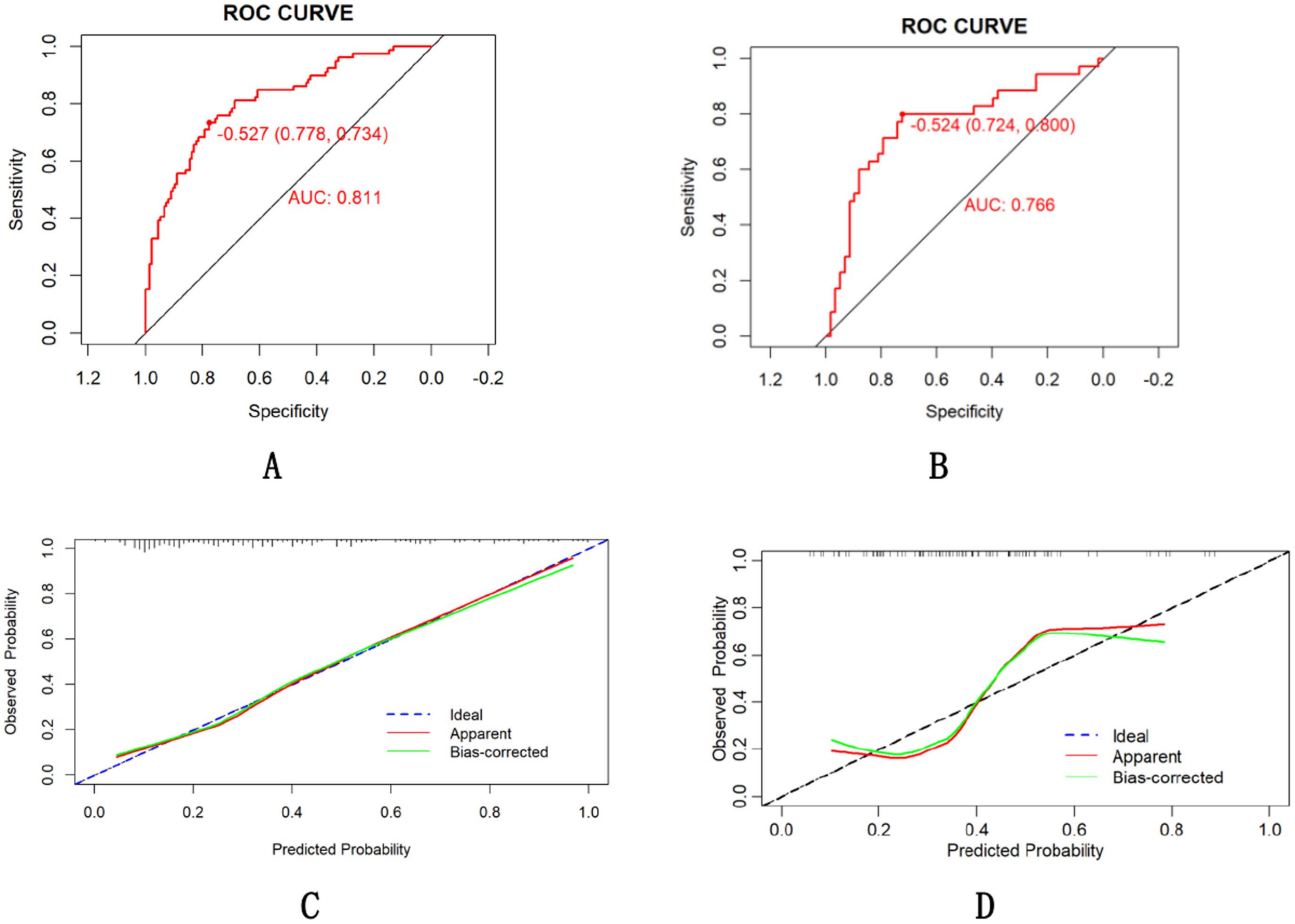
ROC curve of the nomogram for the training **(A)** and validation **(B)** cohorts and calibration curve in the training groups **(C)** and validation groups **(D)**.

The final model achieved an AUC of 0.811 in training groups (accuracy 0.778, sensitivity 0.734, 95% CI 0.751–0.871, Figure 4A) and 0.766 in validation groups (accuracy 0.724, sensitivity 0.800, 95% CI 0.706–0.826, Figure 4B). The calibration curve is shown in Figures 4C,D suggests high consistency, demonstrating that the model’s predicted probabilities are close to the observed actual probabilities [calibration-in-the-large (Intercept = 0.09, *p* = 0.728), spline-based calibration slope (0.50; 95%CI:0.19–0.82)].

### 3.3 Comparison between predict model and established clinical scoring systems

**Figure 5:**
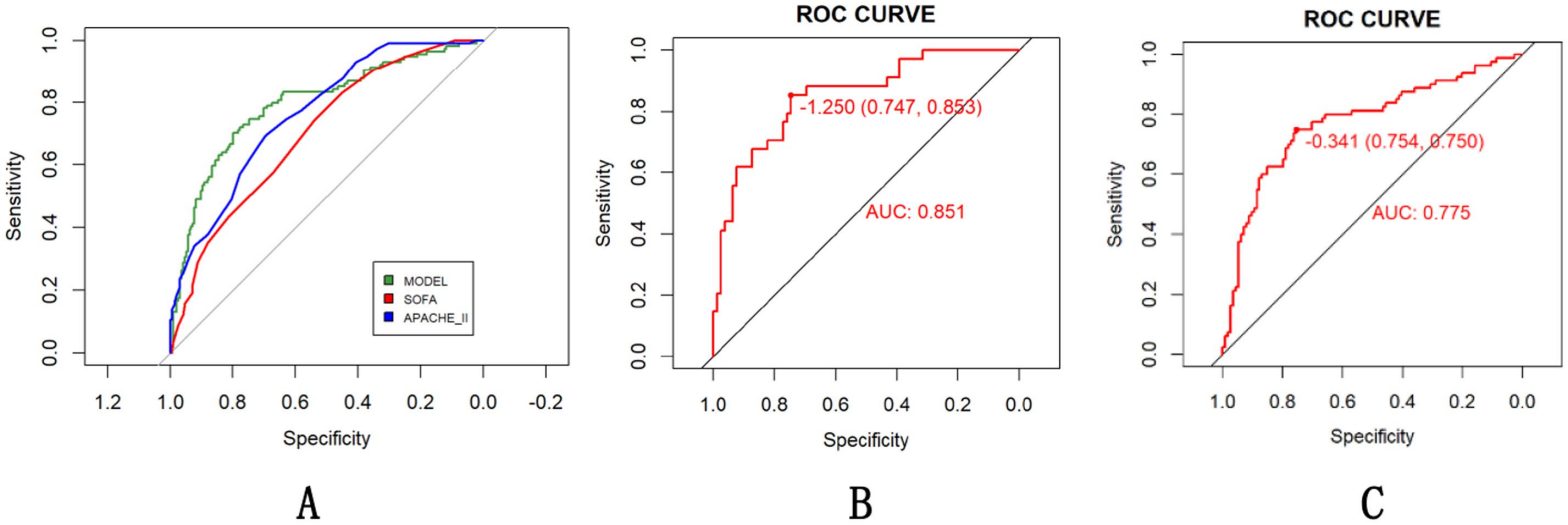
Evaluation of the HS prediction model compared with existing scoring systems **(A)** with subgroup-specific performance [**(B)** EHS, **(C)** CHS] in all population.

Comparative analysis of the model’s predictive efficacy against established clinical scoring systems (SOFA and APACHE II) revealed statistically superior performance in mortality risk stratification. In all populations included in the study, our model achieved an AUC of 0.794, outperforming SOFA (0.703, DeLong’s test, *p* = 0.0008; 95% CI for AUC difference: 0.038–0.144) and non-inferior efficacy compared to APACHE II (0.765, DeLong’s test, *p* = 0.3581; 95% CI for AUC difference: −0.03-0.091) in all patients (Figure 5A). The tool’s robustness across HS subtypes supports broad clinical utility (Figures 5B,C).

**Figure 6:**
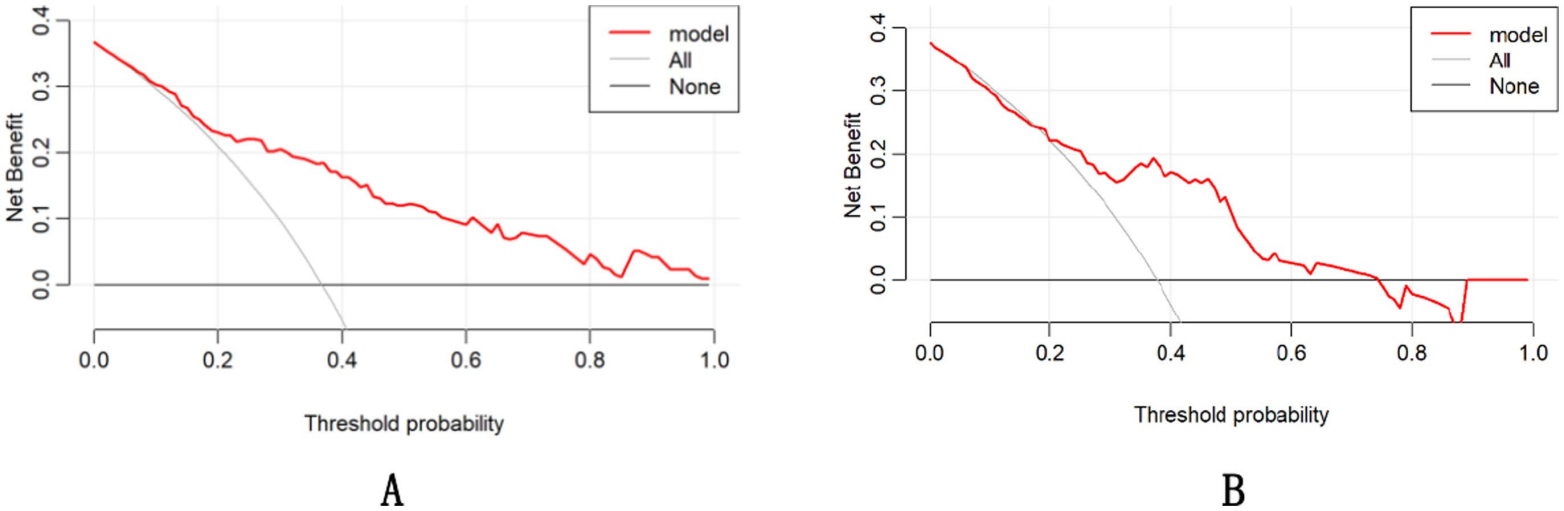
Nomogram decision curve analysis for the training **(A)** and validation **(B)** groups.

**Figure 7:**
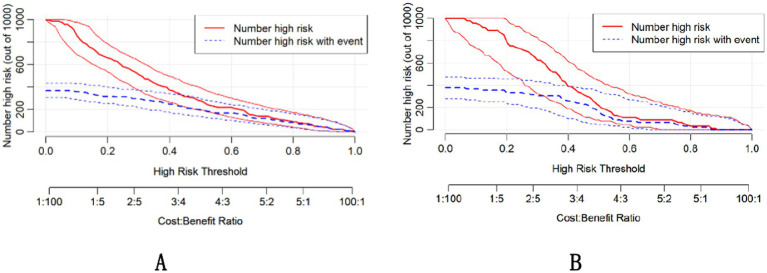
Clinical impact curves for the training **(A)** and validation **(B)** groups.

The DCA demonstrated that the nomogram had a superior overall net benefit across a wide range of practical threshold probabilities (Figures 6A,B). In addition, we plotted clinical impact curves to predict improved probability stratification for a population size of 1,000. The predicted probability coincided with the actual probability in the training and validation cohorts (Figures 7A,B).

## 4 Discussion

**Figure 8:**
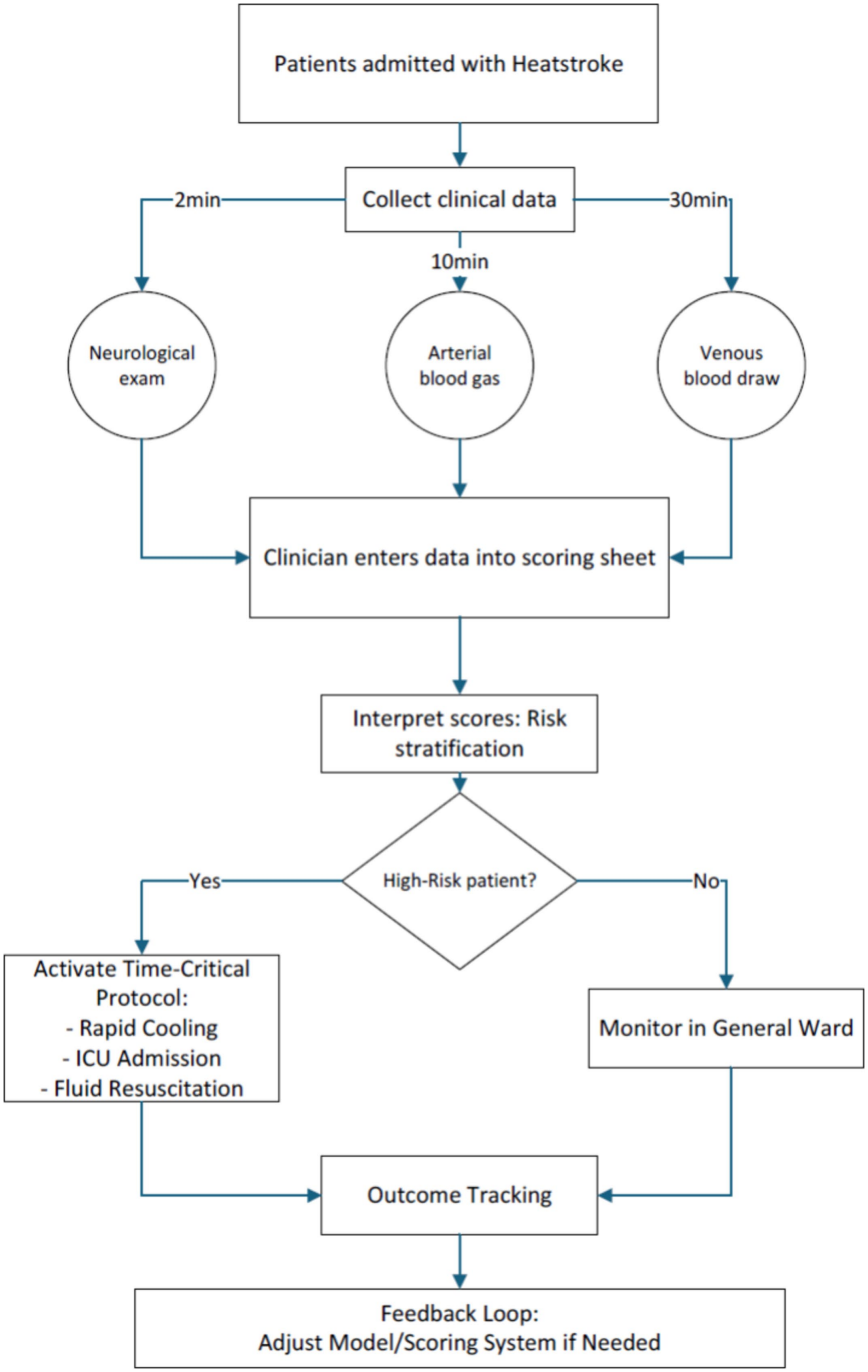
Standardized time-critical data collection protocol for heatstroke patients in emergency settings.

Early prognostic stratification in HS constitutes a critical clinical imperative, given its fulminant progression to multiorgan dysfunction and substantial in-hospital mortality (37% in our cohort). While conventional ICU scoring systems demonstrate epidemiological validity, they remain pathophysiologically agnostic to HS-specific mechanisms – particularly endothelial activation and thermal cytotoxicity cascades. Core temperatures at ED arrival were typically below classical diagnostic thresholds (>40°C), likely reflecting widespread pre-hospital cooling. While this limits temperature’s predictive utility in our model, it highlights the critical need for standardized pre-hospital documentation to enhance future prognostic accuracy. This investigation advances a predictive model integrating admission demographics, physiological parameters (GCS, OI), and biomarker profiles (Cr, FDP) to evaluate hospitalization risks. The resultant nomogram addresses current limitations through two critical innovations: Enhanced biological specificity via biomarkers capturing neuronal thermosensitivity (GCS), renal tubular injury (Cr), hypoxia severity (OI), and disseminated coagulopathy (FDP); Operational practicality through dimensionality reduction from 27 variables to four clinically actionable metrics. Risk stratification can be completed within 30 min using standard ED resources: immediate GCS assessment; arterial blood gas analysis for oxygenation index (OI); and routine laboratory processing of creatinine (Cr) and fibrin degradation products (FDP). Clinicians manually calculate nomogram points without specialized software during initial evaluation (Figure 8).

Furthermore, the developed nomogram demonstrated superior predictive accuracy compared to SOFA scores and non-inferior performance relative to APACHE II, while requiring only admission GCS assessments and routine blood biomarkers (Cr, FDP). This streamlined protocol enabled mortality risk stratification within 30 min of ED arrival - a critical temporal advantage for time-sensitive HS management. Our analysis further identifies key prognostic markers directly informing early resuscitation protocols, establishing a dual-action tool for both rapid triage and targeted intervention in HS. Unlike Wei et al.’s longitudinal model (15) and Wang et al.’s ICU-centric tool (20), our nomogram bridges critical gaps in early HS risk stratification. The 4-parameter framework balances discriminative capacity (AUC > 0.76) with operational feasibility—requiring only: Bedside GCS assessment, Arterial blood gas (OI), Routine labs (Cr, FDP). This enables mortality risk quantification within 30 min, fulfilling unmet needs for rapid ED triage during heat crises.

Our study found that the in-hospital survival outcome of HS patients was significantly correlated with GCS score. Due to the brain’s sensitivity to extremely high temperatures, central nervous system (CNS) injury is considered one of the most early and severe complications of HS. Further, neurological sequelae likely contribute significantly to post hospital mortality (1, 21). In Shi L et al.’s study (22), HS patients with neurological symptoms have a higher risk of ICU mortality (23). In the study by Yang, CNS injury caused by HS was observed that had a significant impact on the prognosis and neurological sequelae of patients, and low GCS score within 24 h of admission may be an independent risk factor for neurological sequelae. HS-induced encephalopathy emerges as an early pathological event preceding multi-organ dysfunction (22), with neurocognitive manifestations constituting the hallmark presentation of HS (2). Thus pathophysiological primacy necessitates emergency evaluation using bedside cognitive assessments enabling rapid severity stratification crucial for judgment of severe HS.

Our study also found that the Cr and OI of HS patients at admission may be related to the prognosis of patients in hospital. HS induces renal compromise through multifaceted pathophysiological mechanisms: not only via dehydration-induced hypoperfusion and reduced renal blood flow, but also through direct thermal cytotoxicity and immune-mediated inflammatory responses that provoke tubular injury (24). These synergistic mechanisms collectively drive serum creatinine alterations, potentially culminating in irreversible nephron damage and adverse patient outcomes. Clinical epidemiology reveals a 35–60% incidence of acute kidney injury (AKI) among HS patients, underscoring the critical need for early biomarkers differentiating transient functional impairment from structural renal damage (25, 26). A retrospective cohort study of 187 EHS cases in Southern China (2008–2019) identified AKI incidence of 44%, with 90-day mortality rates significantly elevated in AKI patients compared to non-AKI counterparts (27% vs. 1%, *p* < 0.001) (27). The impact of elevated serum creatinine on the prognosis of HS patients is considerable, as it increases mortality, especially in those requiring dialysis (28). Besides, impairment in OI may indicate respiratory dysfunction or pulmonary edema in HS patients and exerts substantial influence on in-hospital mortality outcomes. A multinational retrospective analysis encompassing >200,000 ED patients identified OI as a robust predictor of 24-h mortality (29). Notably, OI ≤ 300 mmHg stands as the gold-standard diagnostic criterion for acute lung injury (ALI) (30). Clinical epidemiology reveals ALI prevalence reaching approximately 23, and 75% of patients with ALI eventually die (22). Our study revealed a markedly impaired OI incidence of 74% among HS patients, further corroborating the critical prevalence of respiratory compromise in this population. Historically, few investigations have incorporated arterial blood gas analysis into routine diagnostic protocols for HS, potentially obscuring the recognition of ALI risk in these patients. We advocate for systematic implementation of early arterial blood gas analysis, particularly in HS patients presenting with dyspnea, to comprehensively evaluate systemic physiological status and facilitate timely intervention.

Emerging evidence identifies coagulation dysfunction as a core feature of HS. Consensus guidelines from the Thrombosis and Hemostasis have established that coagulation activation precedes endothelial injury, thrombosis, and hemorrhage, ultimately leading to death (31). FDP, a widely utilized fibrin-related biomarker, serve dual roles of diagnostic and prognostic: both as a diagnostic biomarker for disseminated intravascular coagulation (DIC) identification (19) and a predictive biomarker for venous thromboembolism (VTE) risk stratification. In addition, impaired coagulation function and endothelial injury are also strongly associated with increased risks of cardiovascular morbidity and mortality (32). This pathophysiological primacy mandates emergency coagulation panels within the golden hour of ED presentation, enabling early detection of consumptive coagulopathy critical for guiding mortality risk stratification (33).

The predictive model demonstrated superior discriminative performance to SOFA score and non-inferior prognostic accuracy compared to the APACHE II system. Besides, it is easy to obtain, requiring only the completion of serum creatinine, oxygenation index, FDP and GCS scoring. Implementation of this tool enables precision resource allocation through mortality risk stratification, particularly vital in mass casualty scenarios requiring rapid triage prioritization. In situations where clinicians lack knowledge about the severity of a patient’s condition, this model can provide accurate and prompt prognosis assessments.

Despite including a larger sample size compared to other studies, the inherent limitations and potential data bias of retrospective research cannot be completely eliminated. The retrospective design and regional focus may limit generalizability to populations with distinct climatic or demographic profiles and the observed calibration variation underscores the need for continuous model recalibration during external validation—a priority for our planned multi-regional study. First, although statistical imputation methods were employed to address missing data patterns, the restricted cohort size fundamentally constrained the statistical power for robust validation of secondary endpoints. While our findings demonstrate an expanded sample size relative to prior research, further validation through larger multicenter cohorts remains imperative before clinical implementation can be recommended. Secondly, the heterogeneous disease severity and divergent clinical presentations within our cohort precluded longitudinal tracking of dynamic clinical trajectories. Future studies should assess long-term outcomes (e.g., renal/neurological sequelae) to evaluate the model’s predictive utility beyond in-hospital mortality. Furthermore, external validation in diverse clinical settings is essential before implementing this tool in routine practice. We will prioritize prospective data collection to enhance both sample diversity and temporal resolution of pathophysiological evolution. Given rising HS incidence under escalating global temperatures, future work will prioritize external validation in multi-regional cohorts (e.g., coastal/arid zones) and develop a point-of-care digital calculator. Furthermore, while the multicentric design enhanced epidemiological generalizability, inherent inter-institutional variability in laboratory protocols and operator-dependent analytical techniques may introduce measurement bias. We will systematically address this through standard operating procedure implementation and centralized biomarker assay harmonization in further studies.

## 5 Conclusion

This multicenter study developed and validated a pragmatic prognostic nomogram incorporating four routinely available emergency parameters (serum creatinine, GCS, oxygenation index and fibrin degradation products) to predict in-hospital mortality in HS patients. The model demonstrated superior discriminative performance to SOFA and non-inferior prognostic accuracy compared to the APACHE II while maintaining critical advantages in rapid bedside applicability through elimination of complex physiological calculations.

## Data Availability

The raw data supporting the conclusions of this article will be made available by the authors, without undue reservation.
